# RETRACTION: Study on the Multitarget Synergistic Effects of Kai-Xin-San against Alzheimer's Disease Based on Systems Biology

**DOI:** 10.1155/omcl/9782846

**Published:** 2025-06-10

**Authors:** Oxidative Medicine and Cellular Longevity

RETRACTION: S. Guo, J. Wang, Y. Wang, et al., “Study on the Multitarget Synergistic Effects of Kai-Xin-San against Alzheimer's Disease Based on Systems Biology,” *Oxidative Medicine and Cellular Longevity* 2019, no. 1 (2019): 1–15, https://doi.org/10.1155/2019/1707218.

The above article [1], published online on 31 December 2019 in Wiley Online Library (wileyonlinelibrary.com), has been retracted following the agreement of the journal's Chief Editor Jeanette Vazquez-Vivar; and John Wiley & Sons Ltd.

The retraction has been agreed following an investigation of the concerns raised by *Mycosphaerella arachidis* and *Indigofera tanganyikensis* on PubPeer [1], which identified concerns regarding Figure 4.

Specifically, similarity has been identified between the following panels:

• GR and Hup A
• PR and PO
• Model and AT

In addition, the panel corresponding to PR – treated cells appears similar to the right side of the KXS panel shown in Figure 4d of [2].

As a result of the investigation, the data and conclusions of this article are considered unreliable.

The authors disagree with the wording of this retraction notice.
